# Immunogenicity of Tumor Initiating Stem Cells: Potential Applications in Novel Anticancer Therapy

**DOI:** 10.3389/fonc.2019.00315

**Published:** 2019-04-25

**Authors:** Durga Khandekar, Suneetha Amara, Venkataswarup Tiriveedhi

**Affiliations:** ^1^Department of Biological Sciences, Tennessee State University, Nashville, TN, United States; ^2^Department of Medicine, St. Thomas Hospital-Midtown, Nashville, TN, United States; ^3^Department of Pharmacology, Vanderbilt University, Nashville, TN, United States

**Keywords:** antigens, antibodies, breast cancer, tumor initiating cells, vaccines, immunotherapy

## Abstract

Tumor initiating stem cells (TISCs) are a subset of tumor cells, which are implicated in cancer relapse and resistance to chemotherapy. The metabolic programs that drive TISC functions are exquisitely unique and finely-tuned by various oncogene-driven transcription factors to facilitate pro-cancerous adaptive challenges. While this change in TISC metabolic machinery allows for the identification of associated molecular targets with diagnostic and prognostic value, these molecules also have a potential immunological application. Recent studies have shown that these TISC-associated molecules have strong antigenic properties enabling naïve CD8+T lymphocytes to differentiate into cytotoxic effector phenotype with anticancer potential. In spite of the current challenges, a detailed understanding in this direction offers an immense immunotherapeutic opportunity. In this review, we highlight the molecular targets that characterize TISCs, the metabolic landscape of TISCs, potential antitumor immune cell activation, and the opportunities and challenges they present in the development of new cancer therapeutics.

## Introduction

Drug resistance remains a major challenge in long-term therapeutic success of cancer patients (1). Current anticancer therapies target rapidly dividing cells with the assumption that cancer cells divide at a rate 100–1000 times higher than the normal terminally differentiated cells. Considerable debate exists to explain for the reason behind acquiring the tumorigenicity and heterogeneity of otherwise normal cells. Two non-mutually exclusive theories are well acknowledged in the cancer research field to explain for the tumor heterogeneity (2, 3). One theory proposes the idea that a normal cell becomes a cancer cell due to an acquired or inherited genetic change (referred to as a mutation) giving rise to a clone of cancerous cells. This clone further accumulates genetic changes from environmental or other injuries (second-hit) leading to the evolution of multiple clones, of which, only a few, due to selection pressure and/or due to their ability to escape host immune destruction, ultimately develop into full-fledged cancer. This selection pressure on multiple subclones explains for the tumor heterogeneity (4). Yet another theory, advances an idea that tumors arise from otherwise quiescent progenitor stem cells with a cancer-initiating capability (referred to as tumor initiating stem cells [TISCs]) and upon appropriate external stimulus or an epigenetic change a subset of these stem cells transform to develop full cancers. This proliferative ability leads to expression of markers at various phases of the progression of the stem cells and thus explaining for the final tumor heterogeneity (5). TISCs have been identified in several cancers including breast cancer, brain cancer, and colon cancer (6). TISCs are considered to be resistant to standard chemotherapeutic regimens and play an important role in cancer-relapse. As most of the chemotherapeutic agents target rapidly dividing cells they have minimal effect on quiescent TISCs. From cancer immunotherapy perspective, it is important to note that TISCs display unique heterogeneity with expression of TISC-associated mutated or overexpressed protein commonly referred to as TISC-associated antigens, which open new venues of anti-cancer immunotherapy. Further, TISCs have a distinct metabolic phenotype which leads to overexpression of certain enzymes which could also be utilized for the development of targeted effector immune response. In this review, we will discuss the metabolic phenotypes and molecular *suigeneris* related with TISCs, and then, discuss the possible application of these molecular targets in the development of vaccine and cell based anti-cancer immunotherapeutic tools.

## Phenotypic Differences Between Normal Stem Cells and TISCs

**Figure 1:**
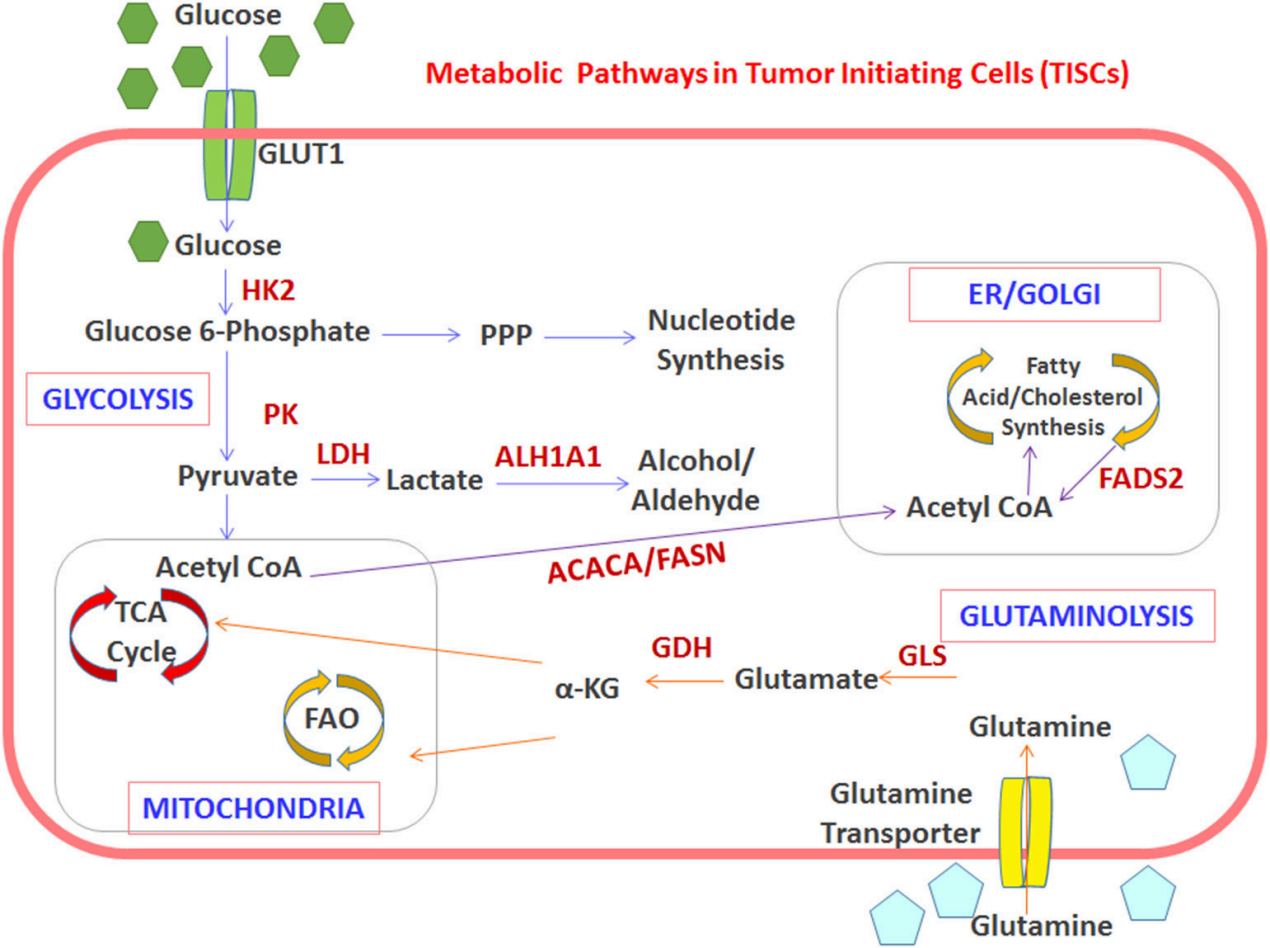
Interplay between TISC metabolism and overexpression of potentially immunogenic antigens. The TISC-associated metabolism enhances the expression of enzymes which offer molecular targets for development of anti-TISC vaccines. Schematic representation of the metabolic switch toward OXPHOS, FA synthesis, and glutaminolysis in TISCs. Upregulated enzymes and pathways are indicated in red. HK2, hexokinase-2; PK, pyruvate kinase; GDH, glutamate dehydrogenase; GLS, glutaminase; ACACA, acetyl-CoA carboxylase; FASN, fatty acid synthase; ALDH1A1, aldehyde dehydrogenase-1A1.

While normal stem cells ([NSCs], such as embryonic stem cells [ESC] and hematopoietic progenitor cells) and TISCs have certain similarities, in that both have the ability to self-renew and differentiate into various organ with histological features, yet, they both have differences in various genetic, morphological and phenotypic features (7). Specifically, there is a stark contrast in the mitochondrial features between NSCs and TISCs, in that mitochondria of NSCs have a lower DNA copy number, poorly developed morphology, and minimal oxidative phosphorylation (OXPHOS) capacity. In contrast, TISCs display increased mitochondrial mass and mitochondrial biogenesis (8). In spite of an increased number of mitochondria, TISCs have been attributed with enhanced glycolytic phenotype, while, terminally differentiated cells were considered to rely mostly on oxidative phosphorylation (OXPHOS) (9, 10) for ATP production. Along with upregulation of glycolysis, TISCs also utilize fatty acid β-oxidation (FAO) and glutaminolysis (Figure 1) which occurs through mitochondrial respiration (11). Interesting, the stem cell features of TISCs such as cell proliferation and migration were inhibited following chemical inhibition of glycolysis, thus suggesting that the glycolytic phenotype of TISCs is needed for their efficient stem-cell functionality (12). When TISCs remain quiescent, their mitochondrial replication and metabolic activity is suppressed (13). However, when quiescent TISCs are subjected to a second-hit by mutation in oncogenes, such as a targeted mutation in a negative regulator of mammalian target of rapamycin (mTOR) complex or tuberous sclerosis complex 1 (TSC1) could lead to a colossal enhancement in the proliferation of TISCs along with upregulation in mitochondrial metabolic activity as evidenced by increase mitochondrial number per cell, elevated production of reactive oxygen species (ROS) and OXPHOS activity eventually leading to tumor relapse (14). These multiple pieces of research evidence suggest that the malignant transition of TISCs from a quiescent to a cancerous state relies on a metabolic switch from glycolytic to mitochondrial-mediated OXPHOS phenotype (15). In addition, modulations in the expression of oncogenic transcription factors, such as Sox2, Oct4, c-Myc, and Klf4, also noted in NSC mediated somatic cell differentiation, are associated with the development of teratomas in murine orthotopic transplant models (16). These data suggest that there is significant overlap in the stem cell signaling mechanisms between somatic cell differentiation and carcinogenesis.

## Unique Metabolic Changes in TISCs

A metabolic comparison between NSCs and TISCs demonstrate that TISCs have elevated Warburg-like glycolytic metabolism with increased glucose consumption, lactate production, and ATP synthesis (17). Research in this area suggests that elevated expression of oncogenes, such as Myc expression, plays a critical role in stem cell functionality and the glycolytic metabolic footprint in some breast cancers (18). A metabolic switch from OXPHOS to glycolysis is noted in TISCs obtained from CD44+basal-like triple negative breast cancer (19). A similar shift to glycolytic metabolism was noted in CD133+TISCs obtained from radio-resistant nasopharyngeal (20) and hepatocellular carcinomas (11). Interestingly, treatment with an inhibitor of glycolysis, 3-bromopyruvate, decreased the stem cell-like functionality and made them more amenable to gemcitabine mediated cytotoxicity in aldehyde dehydrogenase (ALDH) enriched in TISCs obtained from pancreatic ductal adenocarcinomas (21). However, in contrast, CD133+TISCs isolated from certain kinds of glioblastomas and pancreatic cancers displayed an OXPHOS metabolic preference over glycolysis for ATP synthesis (22). This metabolic switch to OXPHOS in TISCs obtained from glioblastomas was shown to be mediated by a growth factor modulating protein, IMP2, which is a known direct enhancer of gene expression in mitochondria and stem-like functionality promoting factors such as CD133, Nanog, and Oct4 (23). Similarly, a metabolic switch to OXPHOS in TISCs from pancreatic cancers was mediated by the upregulation of the transcription factor, PGC-1 α (peroxisome proliferator-activated receptor-γ coactivator-1α) (24). These data suggest that while glycolysis seems to be preferred in TISCs, certain metabolic variations do occur based on the stage and type of cancer which require further study to delineate the molecular bases of these variant metabolic phenotypes.

In the tumor microenvironment, tryptophan metabolism mediated by indoleamine 2,3-dioxygenase (IDO) has a critical immunoregulatory role leading to tumor tolerance. The IDO pathway exerts an immunological role by controlling (inhibiting) inflammation, along with induction of the tolerogenic arm of adaptive immune responses in the tumor *milieu*. Upregulation of IDO depletes the essential amino acid tryptophan leading to activation of the stress-response kinase GCN2, which is a critical molecule involved in sensing amino acid withdrawal (25). GCN2 activation in T cells can inhibit their proliferation, and can skew naive CD4+T cells toward differentiation into a Treg phenotype. In addition, IDO produces the soluble factor, kynurenine, which binds to and activates the aryl hydrocarbon receptor (AhR). Activation of the AhR pathway promotes Treg cell differentiation, and could also induce macrophage polarization to immunosuppressive MΦ2 phenotype. In several small animal cancer models, IDO, which is expressed by tumor cells, was found to stimulate recruitment of Tregs, resulting in the impairment of immune surveillance (26, 27). The use of IDO inhibitors to decrease the overall accumulation of Tregs and enhance tumor regression is an area of intense research in all solid-organ cancers. While enhanced IDO expression was noted in mesenchymal stem cells (28), a similar enhanced expression with downstream immunosuppressive effect is yet to be established in TISCs.

Fatty acid metabolism is an alternative source of energy production used by TISCs. Studies by Wang et al. showed that the lipid metabolism via fatty acid oxidation (FAO) is controlled by the JAK/ STAT3 pathway, which in turn supports breast TISCs and their resistance to conventional chemotherapy (29). These data strongly suggest that JAK/STAT3 pathway inhibition not only affects the self-renewal abilities of breast TISCs, but also blocks the expression of many genes involved in lipid metabolism, such as carnitine palmitoyl transferase 1B (CT1B). Fatty acid oxidation is also known to play a crucial role in maintaining hematopoietic stem cells (HSCs) and memory functionality of CD8^+^ T cells (30). Therefore, FAO inhibition in HSCs has a direct negative impact on their stem cell functionality, whereas, in T cells, this inhibition prevents their phenotypic differentiation (31). These reports suggest that the FAO pathway prioritizes TISC survival over rapid cell proliferation and cancerous differentiation.

## Impact of Tumor Microenvironment on TISCs

The TISCs are further regulated by pro-survival molecules released by other tumor and immune cells in the tumor microenvironment (TME). Hypoxia in the TME seems to be a strong promoter of TISC activity. Various studies have demonstrated that hypoxia in the TME induces expression of HIF-1α, which in turn mediates enhanced expression of down-stream transcription factors with stem-like functionality such as Oct4 and Notch signaling molecules (32, 33). Further, high densities of cancer-associated fibroblasts (CAFs) are also known to promote tumor growth and metastasis (34). A subset of TISCs, CD44+CD90+TISCs, which have been shown to be in direct contact with CAFs in breast cancers are thought to have enhanced tumor invasive functionality (35). Similar metastatic effect was also shown by TISCs following interaction with tumor residing stromal cells in pancreatic cancers (36). Furthermore, in the TME, the interaction of TISCs with stromal cells is suggested to induce epithelial-mesenchymal transition (EMT) resulting in tumor proliferation and metastasis (37). Acquiring EMT properties is a well-known precursor of TISC-induction and cancerous differentiation through upregulation of common pro-cancerous signaling pathways such as the Wnt-signaling mechanism (38, 39). Further, on metabolic front, Wnt-signaling is shown to promote glycolysis and FAO pathways through inhibition of pyruvate dehydrogenase kinase (PDK1), along with upregulation of enzymes such as pyruvate carboxylase, alcohol dehydrogenase, acetyl-CoA carboxylase, and fatty acid synthetase, all of which are associated with the promotion of TISC-mediated EMT (40, 41).

Establishing blood supply to provide oxygen and nutrients is imperative to tumor growth and proliferation. This process of tumor angiogenesis is facilitated by active recruitment of pro-angiogenic endothelial progenitor cells (42). Research on TISCs obtained from glioblastomas have demonstrated that these TISCs have a potential to differentiate into pro-angiogenic endothelial cells potentially leading to vascularization of these tumors. Molecular mechanistic studies have revealed that enhanced expression of vascular endothelial growth factor (VEGF) by tumor cells leads to differentiation of these TISCs into endothelial progenitor cells (43). Further, the cytokine, interleukin-6 (IL-6), is known to induce vascularization in tumors. Inhibition of IL-6-mediated vascular response by inhibition by IL-6 shRNA and IL-6-receptor blocking by tocilizumab demonstrated inhibited cancerous differentiation and growth by CD44+ALDH+TISCs obtained from head and neck squamous cancers (44). These observations suggest that TISCs have angiogenic potential by their ability to differentiate into pro-angiogenic endothelial cells which could be utilized as a therapeutic intervention strategy.

## Potential Impact of TISC-metabolic Phenotype on Innate Immune Responses

Natural killer (NK) cells play a critical role in innate effector immune responses and tumor immune surveillance. While NK cells exert cytotoxicity of tumor cells their effect on anti-TISC immune responses remains undefined. The TISCs have a paradoxical effect toward induction of immune responses. In general, stem-cells, such as TISCs and NSCs, have an inherent immunosuppressive functionality. The TISCs due to their inherent stem-cell functionality have the ability to evade cytotoxic innate and adaptive immune responses. However, in contrast, due to the expression of unique TISC-associated antigens, TISCs could be immunogenic. This intriguing paradoxical effect poses both a challenge and an opportunity toward development of TISC-based anti-cancer immunotherapeutic strategies. A recent study by Ames et al., utilizing orthotopic human tumor implants in immunodeficient murine cancer models, have suggested that NK cells due to their ability to home into non-dividing cells could preferentially target TISCs (45). The authors have utilized the TISC-associated marker ALDH1 to sort stem cells from pancreatic, breast and sarcoma cancer cell lines. The results from this study demonstrated that NK cells co-cultured with these ALDH1 sorted cancer stem cells exerted their cytotoxicity upon ALDH1^high^ cells more effectively than the ALDH1^low^ ones. This preferential targeting was further confirmed on human tumor specimens in single-cell suspension and allogeneic NK cell co-treatment. Based on the molecular mechanistic studies performed in these experiments, the authors conclude that TISCs due to their enhanced surface expression of NKG2D ligands (MICA/B) were able to activate NKG2D receptors resulting in the final NK cell cytotoxic functionality against TISCs. Similar evidence was obtained from other laboratories with studies on TISCs obtained from human colorectal cancers (46). However, in contrast to the above findings, studies with CD133+ brain TISCs demonstrated no significant expression levels of NK cell activating ligands (MICA and MICB), and thus making these glioma TISCs resistant to NK cell-mediated elimination (47). Along these lines, Wang et al., have reported that, in human breast TISCs, aberrant expression of oncogenic miR-20a caused a downregulation of the expression of NKG2D ligands (MICA/B) and eventually resulted in a decreased activation of NK cell receptor NKG2D receptors resulting in possible immune escape of these breast TISCs from NK cell mediated tumor cytotoxicity (28). These data suggest that TISCs probably due to their immature cell differentiation status are not amenable to innate immune mediated tumor elimination response. Taken together, in spite of these conflicting reports, all these interesting observations could provide a strong platform for futuristic NK cell-based anti-cancer immunotherapeutic approaches to eliminate otherwise treatment-resistant TISCs. Furthermore, currently there is only a minimal understanding of the molecular correlation between ALDH1, a protein associated with TISC-associated metabolism, and expression of NKG2D ligands (MICA/B) in TISCs. Mechanistic studies in this direction could shed better light on the potential success of NK cell based immunotherapy.

The immune system due to its double-edged sword nature could exert either inhibitory (tolerance) or stimulatory (cytotoxic) responses on tumor progression. Although, macrophages, due to their phagocytic functionality, are generally considered part of the innate-arm of immunity, tumor infiltrating macrophages display unique two-dimensional plasticity to polarize into two apparently opposite phenotypes (48). While tumor infiltrating macrophages (TAMs) under the influence of various cytokines and chemokines in the TME differentiate into anti-tumor MΦ1 or pro-tumor MΦ2 phenotypes, it is well-recognized that most TAMs display the immunosuppressive MΦ2 phenotype. Cancer cells induce a MΦ2 phenotypic polarization resulting in the secretion of anti-inflammatory and pro-angiogenic cytokines such as, IL-10 and VEGF, which also drive TISC self-renewal by activating cell growth and angiogenesis-related signaling pathways (49). It has been shown that hypoxia in the tumor microenvironment preferentially upregulates OXPHOS and FAO metabolic pathways in the MΦ2 TAMs (50, 51). This causes accumulation of metabolic bi-products such as glutamine, α-ketoglutarate, and succinate resulting in activation of the HIF-1α-mediated cell-self-renewal signaling pathway which is also shown to be critical in TISC functionality (52). Further, TAMs have also been shown to directly enhance carcinogenesis and TISC-dependent chemoresistance through STAT3 transcription factor activation. Other studies have shown a mutually symbiotic relationship between TISCs and MΦ2 TAMs, in that TISCs were considered to play an active role in MΦ2 polarization resulting in inhibition of antigen presentation and anti-tumor cytotoxic CD8+T cell responses (53).

## TISC-associated Antigens

**Figure 2:**
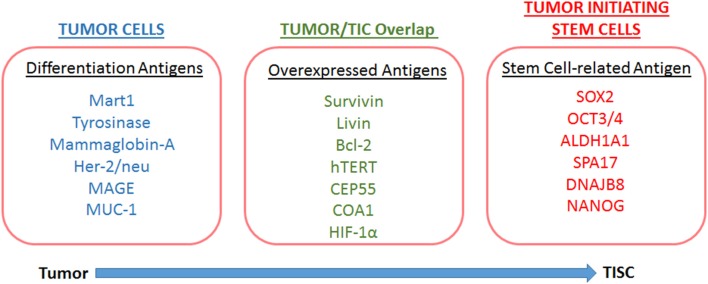
Overview of the antigens associated to tumors and tumor-initiating stem cells.

The ability of a host to recognize TISCs as non-self and mount an efficient effector immune response would be critical for the development of novel TISC based immunotherapeutic strategies. Antigen expression is essential for recognition by naïve T-lymphocytes and efficient induction of a CD8+T lymphocyte (CTL) response (54). Unique antigen expression profiles in TISCs have been reported in several malignancies. TISC-associated antigen profiles (Figure 2) could be a result of either over-expression of antigen, expression of a differentiation antigen, or mutation of normal somatic protein resulting in neo-antigens (55). As TISCs are immature forms of cancer cell differentiation, the differentiation antigens are not generally considered as suitable targets for the development of TISC-associated immunotherapeutic strategies. TISCs express several overexpressed antigens, such as CEP55, COA1 etc., which are also over-expressed in normal stem cells (56, 57). Although all over-expressed antigens might not be strong immunotherapeutic targets, certain other types of overexpressed antigens, such as ALDH1A1 (58), survivin, livin, and Bcl-2 (59, 60), have been reported in TISCs. These antigens while ubiquitous and expressed in minimal quantities in normal organs, are over-expressed in TISCs and play a critical role in tumorigenesis. Along with this, organ-associated over-expression of antigens such as, hTERT in CD44+ breast cancer TISCs (61), HER2 proto-oncogene in glioma TISCs (62), CEP55, and COA-1 (63) in colon TISCs is well-established. These overexpressed antigens could be novel targets for the development of TISC-associated immunotherapeutic strategies.

**Figure 3:**
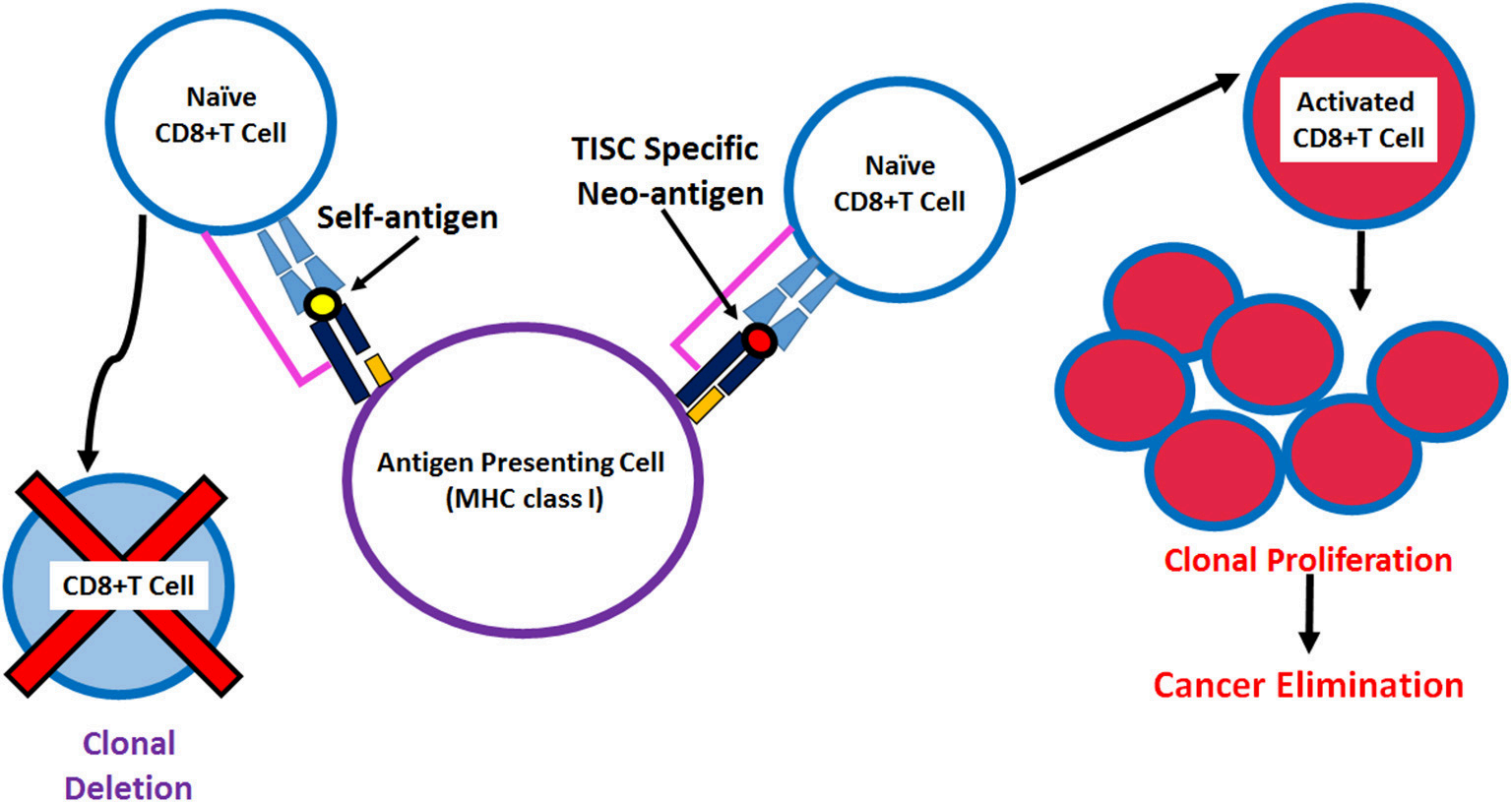
Schematic representation of the antigen presentation of neoantigen derived immunodominant epitopes by MHC class I molecules to activate CD8+T cell against tumor initiating stem cells.

**Figure 4:**
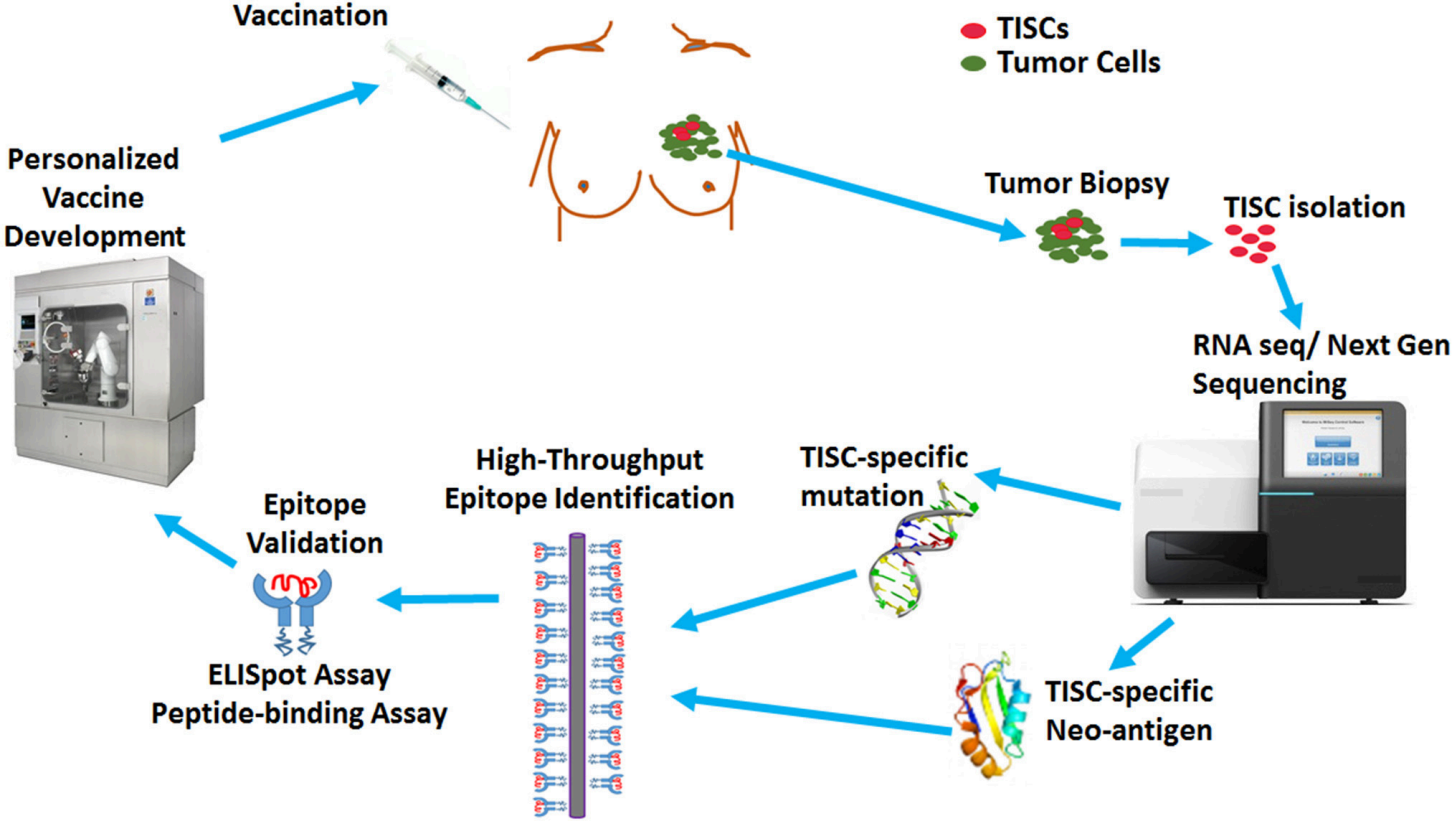
Overview of TISC-based cancer vaccine design. The TISCs isolated from the cancer patients will be subjected to mRNA-based next generation sequencing to identify TISC-associated overexpression or TISC-associated non-synonymous somatic mutations. The identified molecular targets will be validated by *in silico* prediction algorithms and high throroughput MHC class I binding technologies to rank a list of high-valued candidate epitopes, which are further validated for their autologous activation of naïve CD8+T cells by IFN-γ ELISpot. These validated epitopes will be utilized by biotechnology companies for development of novel personalized vaccines and dendritic cell based immunotherapies.

Expression of neo-antigens in TISCs is a result of genomic DNA mutations resulting in the production of tumor-associated antigens (64, 65). As the antigenic peptide epitopes of these neo-antigens are not significantly affected by central T-cell tolerance compared with non-mutated self-antigens (Figure 3), these neo-antigens could offer attractive peptide-base anti-cancer vaccine strategies (66). With the advent of mass spectrometry and next-generation exome sequencing tools, neo-antigens are expected to play a critical role in personalized cancer medicine (67, 68). However, these *in silico* neo-antigen identification techniques should be supplemented by extensive bench-work studies to determine if the mutation resulting in a neo-antigen is a driver mutation with cell growth advantage or passenger mutation with no cell growth advantage (Figure 4). Further, expression of neo-antigens depends upon the actual transcription and translation of the mutated gene, thus, limiting the final number of neo-antigens in TISCs and compromising the potential development of immunotherapeutic strategies (69).

Activation and differentiation of naïve CD8+T lymphocytes to cytotoxic effector cells requires efficient loading and membrane surface presentation of immunodominant epitopes by HLA class I (70). However, efficient presentation of TISC-associated antigenic peptides is reduced due to downregulation of surface expression of HLA class I molecules on mature cancer cells. This helps TISCs escape host immune surveillance. Loading of MHC class I molecules with TISC-associated antigenic peptides requires intact antigen presenting machinery (APM) comprised of the proteasome complex needed for cleavage of antigenic proteins, transport of cleaved antigenic peptides into endoplasmic reticulum (ER) through transporters associated with antigen processing-1 and−2 (TAP1 and TAP2), further intra-ER cleavage of peptides to appropriate length to be loaded on to MHC class I and β2-microglobulin (B2M) complex by endoplasmic reticulum aminopeptidase associated with antigen processing (ERAAP), and several chaperone molecules, such as tapasin, calreticulin, ERp57, and calnexin, needed for efficient antigenic peptide loading onto MHC class I (71). In contrast to tumor cells, studies by Chikamatsu et al. report that CD44+TISCs demonstrated expression levels of the molecules involved in antigen presentation machinery (APM), such as LMP2, LMP7, TAP1 etc., equivalent to that of CD44 negative stromal non-stem cells. These data clearly suggest that TISCs have the ability to present immunodominant epitopes by HLA class I molecules and, therefore, could potentially be killed by TISC-associated antigen-specific cytotoxic response by CD8+T lymphocytes (CTL) (72). Interestingly, HER2-specific peptide vaccination reduced ALDH positive breast cancer associated TISCs in MMTV-PyMT murine transgenic breast cancer models, suggesting a potential immunotherapeutic treatment strategy against treatment resistant TISCs (73). However, there seems to be varying evidence for the expression of HLA class I molecules on TISCs based on the tissue origin. For example, studies on TISCs derived from melanoma demonstrated reduced HLA class I expression suggesting that HLA class I expression in TISCs depends on the tumor type (74). Furthermore, even within the same tumor type, two apparently conflicting reports came from two different groups. While one research group showed enhanced HLA class I expression in glioblastoma multiform (GBM) derived TISCs isolated as sphere-forming cells, while other research group working on similar GBM derived TISCs as sphere-forming cells reported lower HLA class I expression (75). Further, analysis on *in vitro* 3D cell cultures of TISCs derived from GBM has demonstrated reduced expression of immuno-stimulatory CD80 and CD86 molecules, while, there was an enhanced expression of the immuno-inhibitory molecule, PD-L1, and secretion of the immunosuppressive cytokine, IL-10 (76). This immunosuppressive profile of TISCs might contribute to creating an immune-suppressive tumor microenvironment resulting in tumor immune escape. The difference in HLA expression and ability to mount adaptive immune responses amongst various cancer cell lines could be from a difference in TISCs isolation methodology and/or varying organ-associated origin of TISCs which requires further investigation.

## TISC Antigen-Based Vaccines and Monoclonal Antibodies

The enzymes overexpressed to mediate TISC-skewed metabolic pathways are considered to be good antigenic targets for development of anti-TISC-based immunotherapies. For example, an isoform of aldehyde dehydrogenase (ALDH1A1) promotes cell-survival by reducing the intracellular cytotoxic oxidative damage mediated by oxidation of aldehydes to carboxylic acids (77). Enhanced ALDH1A1 enzymatic activity was noted in TISCs obtained from various solid organ tumors. In addition, ALDH1A1 is considered to detoxify metabolic bi-products from chemotherapy and thus conferring cancer resistance in ALDH1A1^high^ cancer cells (78). Dylla et al., have demonstrated that shRNA based knock-down of ALDH1A1 mRNA significantly increased the chemo-susceptibility of TISCs obtained from colon cancer to cyclophosphamide therapy (79). Several groups have demonstrated that ALDH is an attractive antigenic target for induction of anti-cancer adaptive immune responses. Visus et al. have utilized immunodominant epitopes derived from ALDH to generate cytotoxic effector CD8+T cells specifically against ALDH producing cancer cells (35, 58). Interestingly, autologous dendritic cells pulsed by immunodominant epitopes derived from ALDH were utilized for *in vitro* stimulation of patients' CD8+T cells to activate them into cytotoxic cells. Similarly, in preclinical immunocompromised murine cancer models adoptive transfer of CD8+T cells activated by ALDH-epitope pulsed dendritic cells reduced the tumor growth kinetics of human cancer xenografts (80). Taken together, these data provide compelling evidence for the future utilization of ALDH as TISC-associated antigenic target to develop novel peptide and DNA-based vaccine strategies. Further, *in vitro* activation of dendritic cells (DCs), by antigenic epitopes and eventual adoptive transfer of these DCs is considered a promising anti-cancer vaccination strategy. Phuc and colleagues reported that breast TISC-associated antigen derived DC vaccine could lead to migration of adoptively transferred pulsed-DCs to the spleen and activation of naïve CD8+T cells and induce anti-tumor cytotoxicity (81). These observations, although require further study, nevertheless, strongly suggest the futuristic application of TISC-associated antigen pulsed DC-based immunotherapy.

A TISC-associated membrane protein, CD44, is a 90 kDa glycosylated type-1 p-glycoprotein which is involved in several stem cell-like functions such as self-renewal, cell division, anti-apoptosis, and distant metastasis (82). Targeting TISCs by inhibiting CD44 signaling with blocking monoclonal antibodies (mAb) has emerged as a promising anti-cancer therapy. Seiter et al., have shown a reduced proliferation and lung metastases of pancreatic adenocarcinoma in small animal tumor model following treatment with mAbs against CD44v, a splice variant ofCD44 (83). Following a similar approach, Jin et al., have demonstrated that treatment with CD44 mAb specifically eradicated AML TISCs in immunodeficient murine cancer model (84). Further, there was an 80% reduction of BxPC3 pancreatic tumor xenografts following administration of a humanized CD44-specific mAb (85). Human clinical trials with anti-CD44 mAb seem like a promising immunotherapeutic approach (86). Similarly, other surface markers such as CD133, survinin, Her-2, and CT proteins are targeted by mAbs to promote tumor regression in various preclinical models.

## Conclusion

Several TISC-based immunotherapeutic approaches are under development in various stages of preclinical studies. As outlined in this review article, a careful and more exhaustive genetic and metabolic understanding of TISC-associated phenotypes is critical to develop novel TISC based immunotherapies. Various components within the tumor microenvironment such as tumor cells, infiltrating immune cells, and supporting stromal cells impact the TISC metabolism. This unique metabolic profile leads to upregulation of certain enzymes and proteins such as ALDH1, CEP55, IDO COA1 etc., which can be utilized for development of vaccine based anti-cancer immunotherapy. Further investigation of TISCs and the immunosuppressive phenotype caused by the tumor microenvironment will provide opportunities to not only achieve a more comprehensive understanding of tumor biology but also develop specific medical therapies to target the weaknesses underlying tumor development and attack tumor cells in more effective ways. For example, various preclinical studies have clearly demonstrated that combination immunotherapies such as vaccines, Treg depletion, or immune checkpoint blockade, together with chemotherapy have more profound outcomes compared to conventional chemotherapy alone. Further, application of novel technologies and high-throughput platforms would be needed to identify TISC-associated neoantigens for future development of anti-cancer vaccine strategies. These TISC-associated strategies could also be combined with other immunotherapies such as immune-check-point inhibitor (PD1/CTLA-4) for enhanced anticancer therapeutic success.

## Author Contributions

DK, SA, and VT participated in manuscript drafting, and revision of this review article.

### Conflict of Interest Statement

The authors declare that the research was conducted in the absence of any commercial or financial relationships that could be construed as a potential conflict of interest.
